# Hand Hygiene Knowledge and Perception among the Healthcare Workers during the COVID-19 Pandemic in Qassim, Saudi Arabia: A Cross-Sectional Survey

**DOI:** 10.3390/healthcare9121627

**Published:** 2021-11-24

**Authors:** Adil Abalkhail, Ilias Mahmud, Fahad A. Alhumaydhi, Thamer Alslamah, Ameen S. S. Alwashmi, Divya Vinnakota, Russell Kabir

**Affiliations:** 1 Department of Public Health, College of Public Health and Health Informatics, Qassim University, Buraydah 52741, Saudi Arabia; abalkhail@qu.edu.sa (A.A.); 4037@qu.edu.sa (T.A.); 2 Department of Medical Laboratories, College of Applied Medical Sciences, Qassim University, Buraydah 52571, Saudi Arabia; f.alhumaydhi@qu.edu.sa (F.A.A.); aswshmy@qu.edu.sa (A.S.S.A.); 3 School of Allied Health, Faculty of Health, Education, Medicine and Social Care, Anglia Ruskin University, Chelmsford CM1 1SQ, UK; drdivya0424@gmail.com (D.V.); russell.kabir@aru.ac.uk (R.K.)

**Keywords:** healthcare workers, hand hygiene, hospital-acquired infection, Saudi Arabia

## Abstract

Hand hygiene is among the most important factors of infection control in healthcare settings. Healthcare workers are the primary source of hospital-acquired infection. We assessed the current state of hand hygiene knowledge, perception, and practice among the healthcare workers in Qassim, Saudi Arabia. In this cross-sectional study, we used the hand hygiene knowledge and perception questionnaire developed by the World Health Organization. Knowledge and perceptions were classified into good (80–100%), moderate (60–79%), and poor (<60% score). The majority of the healthcare workers had moderate knowledge (57.8%) and perception (73.4%) of hand hygiene. Males were less likely to have moderate/good knowledge compared to females (OR: 0.52, *p* < 0.05). Private healthcare workers were less likely (OR: 0.33, *p* < 0.01) to have moderate/good perceptions compared to the government healthcare workers. Healthcare workers who received training on hand hygiene were more likely to have good/moderate perception (OR: 3.2, *p* < 0.05) and to routinely use alcohol-based hand rubs (OR: 3.8, *p* < 0.05) than the ones without such training. Physicians are more likely (OR: 4.9, *p* < 0.05) to routinely use alcohol-based hand rubs than technicians. Our research highlighted gaps in hand hygiene knowledge, perception and practice among healthcare workers in Qassim, Saudi Arabia and the importance of training in this regard.

## 1. Introduction

Hand hygiene is among the most important factors of infection control in a healthcare setting. Healthcare workers are often responsible for transmission of pathogens from one patient to the other through their contaminated hands [1]. Healthcare-associated infections are a serious burden to the healthcare settings. A recent meta-analysis reported that USD 9.8 billion was spent yearly by the hospitals in the USA to combat different types of hospital-acquired infections [2]. Reduced hand hygiene compliance is considered to be a global problem and compliance also differs among different healthcare professionals [3].

Hand hygiene is defined as the primary measure known to be effective in preventing healthcare-associated infections and preventing the spread of antimicrobial resistance [4]. Washing hands either with water and soap or using an alcohol-based hand rub is the most cost-effective public health measure that can prevent healthcare-associated infections [1,5]. In 2009, the WHO issued guidelines concerning hand hygiene procedures to reduce the prevalence of hospital-associated infections [6]. Though hand-washing is a simple procedure, some healthcare workers are reluctant to adopt the recommended hand hygiene practices. Poor compliance of the healthcare workers in following recommended hand hygiene procedures is associated with a lack of adequate knowledge, awareness, and attitude towards hand hygiene [6,7]. 

Healthcare workers have the responsibility to prevent cross-contamination, especially nurses. Nurses are more likely to be responsible for the transmission of infection-causing microorganisms in hospitals since they are higher in number and are the people who mostly come in contact with patients and contaminated objects [8,9]. Nursing interventions require direct contact with patients, hence becoming an avenue for pathogen transfer if hand hygiene is not followed correctly [8,10].

According to the WHO, an estimated 1.4 million people globally are affected by healthcare-associated infections at any time [6]. There are many consequences associated with healthcare-associated infections—prolonged stay in the hospital, disability, higher healthcare cost for patients and families, increased morbidity and mortality, and increased resistance to antibiotics. All these in turn increase the financial burden on the health system [11]. Similar to the rest of the world, Saudi Arabia has a great concern about healthcare-associated infections. According to a study conducted in a Saudi Arabian military hospital in the year 2004 about nosocomial infections, among 1382 patients who developed an infection following hospital admission, 48.3% had nosocomial infections and of all the healthcare-associated infections reported, there were respiratory tract infections (32.3%), urinary tract infections (25.3%), blood streem infections (18.6%), and surgical site infections (12.9%) [12]. Furthermore, another study conducted in hospitals in Taif, Saudi Arabia from 2010 to 2011 states the smilar findings [13]. Therefore, healthcare-associated infections are considered a significant public health concern for patients, healthcare workers, and the health system [6]. In this context, the aim of this study was to evaluate the hand hygiene knowledge, perception, and practices of the healthcare workers in Qassim, Saudi Arabia.

## 2. Methods

### 2.1. Study Design, Settings, and Sampling

We did a cross-sectional online survey of healthcare workers between October 2020 and March 2021 in Buraidah and Ar-ras cities of the Qassim region, Saudi Arabia. An online structured survey form which was developed on the Google platform was disseminated to the healthcare workers in these two cities through our professional and social networks using emails and WhatsApp. The study purpose and title were clearly indicated on the front page of the online form, and the participants were requested to avoid multi-registration. A total of 301 healthcare workers completed the online survey. Ethical clearance for this research was obtained from the regional research ethics committee, Qassim, Saudi Arabia. Informed consent was obtained from the participants. This research has anonymized data and the anonymized dataset was kept on a password-protected laptop and was only accessible to the researchers.

### 2.2. The Instrument

Our structured questionnaire had three parts. The first part collected healthcare workers’ socio-demographic information. The second and third parts collected information on healthcare workers’ knowledge and perception of hand hygiene, respectively. To assess healthcare workers’ knowledge and perceptions on the essential aspects of hand hygiene, we used the hand hygiene knowledge [6] and perception questionnaire [14] for healthcare workers developed by the WHO.

### 2.3. Variables

To assess hand hygiene knowledge, for each correct response participants were given 1 point and 0 points for each wrong response. The overall knowledge was categorized into good (80–100% score), moderate (60–79%), and poor if the score was less than 60%. We assessed perceptions of the healthcare workers on essential aspects of hand hygiene using 10 questions. Participants’ responses on each of these 10 questions were assessed with a five-point (0–4) Likert-type scale—the higher the score better the perceptions. We computed the total perceptions score and the overall perception was categorized into good (80–100% score), moderate (60–79%), and poor if the score was less than 60%. For the multivariable logistic regression analyses, we categorized knowledge and perceptions as moderate or good (60–100% score) and poor (<60% score). We assessed the hand hygiene practice of the healthcare workers using a single item—whether or not the healthcare worker routinely used an alcohol-based hand rub while working. 

### 2.4. Analysis

We did a descriptive analysis of the knowledge, perceptions, and practice questions. We reported frequency and percentages for each of the knowledge, perceptions, and practice items. We classified healthcare workers’ knowledge and perceptions into good, moderate, and poor. We reported the number and proportions of the healthcare workers in each of these categories. 

We did multivariable logistic regression analysis to investigate the factors associated with moderate to good knowledge, moderate to good perceptions, and routinely using an alcohol-based hand rub. For multivariable logistic regression analyses, we reported odds ratio (OR) with a 95% confidence interval (CI). In addition, we reported corresponding *p* values. A *p*-value of <0.05 was considered statistically significant. 

## 3. Results

Table 1 shows the background characteristics of the healthcare workers participated in this study. The background characteristics of the participants reveal that around 69.8% of the healthcare workers were from the government health facilities, whereas only 30.2% were from the private health facilities. Among all the healthcare workers, the majority of them were males (55.8%) when compared to females (44.2%). Approximately 57.5% of healthcare workers belonged to the age group of 20–34 years, followed by the 35 or over age group (42.5%). According to the nationality, about 70.1% were Saudi and the remaining 29.9% were non-Saudi. The healthcare workers with graduate-level of qualification were 86.7% and only 13.3% had a postgraduate level of qualification. 

Table 2 presents hand hygiene knowledge of healthcare workers in Qassim, Saudi Arabia. When participants were asked about their knowledge on hand hygiene issues, about 64.5% of the participants revealed that the main routes of cross-transmission between patients are when their hands are not clean. Approximately 28.2% of the healthcare workers reported that germs already present on or within the patient are the main source of germs responsible for healthcare-associated infections. About 94.4% of the healthcare workers shared that hand hygiene action prevents transmission of germs before touching a patient. Additionally, a vast majority of the participants, 90.4% and 88%, respectively said that damaged skin and artificial fingernails should be avoided as associated with a likelihood of colonization of the hand with harmful germs. Overall, about 57.8% of healthcare workers provided 60 to 79% correct responses.

Table 3 shows the perception of healthcare workers about hand hygiene. About 63.5% reported that the impact of healthcare-associated infection on patient’s clinical outcome is very high whereas only 0.3% reported as very low. Around 67.8% of their perception is very high about the effectiveness of hand hygiene in preventing healthcare-associated infection whereas 0.7% of low perception. Among all patient safety issues, 65.4% of the healthcare workers consider that hand hygiene should be of very high priority within management priorities at the institution. However, 1.7% of them consider it a very low priority. To increase hand hygiene permanently in health facilities, a few actions should be taken such as leaders and senior managers supporting and openly promoting hand hygiene, which healthcare workers reported as very effective (55.8%), whereas there are few who felt it as not effective (1.7%). While coming to alcohol-based hand-rub availability at each point of care in healthcare facilities, around 51.5% of the health care workers opined it as a very effective action in contrast 1.7% of them opined as a not-effective measure. About 0.7% of the healthcare workers saw hand hygiene posters displayed as reminders at the point of care as ineffective, whereas approximately 50.8% thought it was very effective. According to the healthcare workers’ opinion about receiving education on hand hygiene to increase hand hygiene permanently in their health facility, 1% of healthcare workers said it was not effective, however, the majority of them believed it was very effective (55.8%). Similarly, healthcare workers thought that clear and simple instructions for hand hygiene making visible for every healthcare worker (55.8%) and healthcare workers regularly receiving the results of their hand hygiene performance are very effective (41.5%). Another measure to increase hand hygiene was patients being invited to remind healthcare workers to perform hand hygiene, only 28.6% of healthcare workers saw this as very effective whereas 4% of their perception for this measure was that it was ineffective.

Table 4 presents the determinants of hand hygiene knowledge, perception, and practice among healthcare workers in the Qassim region, Saudi Arabia. We found no evidence of a statistically significant association between hand hygiene knowledge and demographic and professional variables apart from gender. After adjusting for the effect of all demographic and professional variables included in the model, we found that males were 48% less likely to have moderate to good knowledge compared to females (OR: 0.52; 95% CI: 0.28–0.98; *p* < 0.05).

Regarding the perception of hand hygiene, we found evidence of a statistically significant association with education, types of healthcare facilities, and training. We found that healthcare workers with postgraduate level education were less likely to have a moderate or good perception (OR: 0.27; 95% CI: 0.09–0.80; *p* < 0.05). We also found that healthcare workers from the private facilities were 67% less likely (OR: 0.33; 95% CI: 0.15–0.73; *p* < 0.01) to have moderate/good perception compared to the healthcare workers from the government facilities. Regarding training on hand hygiene, we found that healthcare workers who received training on hand hygiene were 3.2 times more likely (95% CI: 1.24–8.21, *p* < 0.05) to have a good/moderate perception about hand hygiene compared to the ones who did not receive training. 

Our multivariable logistic regression analysis results suggest evidence of statistically significant association (*p* < 0.05) between routinely using alcohol-based hand rubs and type of profession, level of education, and training. Physicians were 4.9 times more likely (95% CI: 1.06–22.75; *p* < 0.05) to routinely use alcohol-based hand rub than the technicians, after adjusting for the effect of other variables. In addition, we found that healthcare workers who received training on hand hygiene were 3.8 times more likely (95% CI: 1.2–11.67; *p* < 0.05) to routinely use alcohol-based hand rub than the ones who did not receive such training.

## 4. Discussion

Our study aimed to assess the hand hygiene knowledge, perception, and practice among the healthcare workers in Qassim, Saudi Arabia. The study findings revealed that about 58% of the healthcare workers have moderate knowledge of recommended hand hygiene, while 41% have poor knowledge. Moderate knowledge among healthcare workers in hospital settings is also reported in studies conducted in North-Central Nigeria, Iran, India, and Pakistan [15,16,17,18]. This could be a reason for concern as some studies also found that hand hygiene practice remains low despite a good amount of knowledge [19].

The study participants also implied that wearing artificial jewellery, fingernails, and damaged skins could be the sources of spreading germs in hospital settings. A similar result was reported by Suen et al. (2020) in Hong Kong [20], and Maheshwari et al. in Bhopal, India (2014) [7].

Our multivariate regression analysis revealed an association between knowledge on hand hygiene and the gender of the healthcare worker. Our study has shown that female healthcare workers have comparatively better knowledge of hand hygiene than male healthcare workers. This finding agreed with a cross-sectional study in Hong Kong, where female healthcare workers demonstrated significantly higher knowledge than male healthcare workers [20]. The study findings also reported that healthcare workers with post-graduation education are less likely to have moderate or good hand hygiene perception. Research has shown educational intervention is a significant predictor of good hand hygiene compliance and hand hygiene education courses are needed to improve the competency of infection control issues among healthcare workers [21]. A qualitative study conducted in Iran among healthcare workers found that appropriate education positively affects hand hygiene behavior and attitude [22]. We found that the healthcare workers who attended training activities on hand hygiene are more likely to have a better perception of the issue. This is consistent with other qualitative and quantitative research findings conducted in Turkey and Uttarakhand, India [19,23]. The importance of repeated hand hygiene training for healthcare workers has also been emphasized to reduce hospital-associated infections [7].

Previously it has been reported that physicians’ perceptions and perceived effectiveness of hand hygiene to some extent vary from other healthcare professionals [24]. The physicians of this study also have shown similar characteristics. They are more regularly using alcohol-based hand rubs to protect themselves from germs compared to other professional healthcare workers. This is also congruent with a research done in Pakistan by Ahmed et al. in 2020 [15]. However, evidence from a teaching hospital in India shared that understanding the importance of hand hygiene is an essential driving factor among healthcare professionals to regularly maintain the practice [25]. 

In this study, it is evident that moderate knowledge among healthcare workers is the main barrier to maintaining good hand hygiene practice at the workplace. More repeated training courses and a culture of promoting good hand hygiene practice should be promoted frequently. The main limitation of the study is that the data has been gathered from the healthcare workers in two Qassim cities using an online survey, which might limit the generalization of the study findings. Additionally, this study utilized self-reported data and therefore we cannot rule out the possibility of information bias. Future studies incorporating observational data and documentary analysis are necessary to investigate what is happening in real-world practice. However, the findings of the study would be useful for the hospital authorities to take extra precautions and they should make necessary arrangements to provide extra support on a regular basis to the healthcare workers working at the front line to deal with any public health emergency that arises.

## Figures and Tables

**Table 1:** Characteristics of the participants.

Characteristics	Frequency	Percent
**Type of health facility**		
Governmental	176	69.8
Private	76	30.2
**Gender**		
Female	133	44.2
Male	168	55.8
**Age**		
20–34 y	173	57.5
35 or over	128	42.5
**Nationality**		
Saudi	211	70.1
Non-Saudi	90	29.9
**Qualification**		
Graduate level	261	86.7
Postgraduate level	40	13.3

**Table 2:** Hand hygiene knowledge of healthcare workers in Qassim, Saudi Arabia.

Knowledge Items (Correct Response)	Correct Response	
	Frequency	Percent
**The main route of cross-transmission of potentially harmful germs between patients in a healthcare facility**		
Healthcare workers’ hands when not clean (Yes)	194	64.5
Air circulating in the hospital (No)	285	94.7
Patients’ exposure to colonized surfaces (No)	223	74.1
Sharing non-invasive objects (stethoscopes, pressure cuffs, etc.) between patients (No)	288	95.7
**The most frequent source of germs responsible for healthcare-associated infections**		
The hospital’s water system (no)	289	96.0
The hospital air (no)	276	91.7
Germs already present on or within the patient (yes)	85	28.2
The hospital environment (surfaces) (no)	122	40.5
**Hand hygiene actions prevent transmission of germs to the patient**		
Before touching a patient (yes)	284	94.4
Immediately after a risk of body fluid exposure (no)	41	13.6
Immediately before a clean/aspetive procedure (yes)	258	85.7
After exposure to the immediate surroundings of a patient (no)	45	15.0
**Hand hygiene actions prevent transmission of germs to the healthcare worker**		
After touching a patient (yes)	272	90.4
Immediately after a risk of body fluid exposure (yes)	272	90.4
Immediately before a clean/aseptic procedure (no)	43	14.3
After exposure to the immediate surroundings of a patient (yes)	261	86.7
**Alcohol-based hand-rubs versus handwashing with soap and water**		
Hand rubbing is more rapid for hand cleansing than handwashing (true)	197	65.4
Hand rubbing causes skin dryness more than handwashing (false)	214	71.1
Hand rubbing is more effective against germs than handwashing (true)	136	45.2
Handwashing and hand rubbing are recommended to be performed in sequence (false)	233	77.4
**The minimal time needed for alcohol-based hand rub to kill most germs on hands (20 seconds)**	241	80.1
**Type of hand hygiene method required in the following situations**		
Before palpation of the abdomen (rubbing)	125	41.5
Before giving an injection (rubbing)	207	68.8
After emptying a bedpan (rubbing)	89	29.6
After removing examination gloves (rubbing)	129	42.9
After making a patient’s bed (rubbing)	110	36.5
After visible exposure to blood (washing)	183	60.8
**Should be avoided, as associated with a likelihood of colonization of hand with harmful germs**		
Wearing jewellery (yes)	218	72.4
Damaged skin (yes)	272	90.4
Artificial fingernails (yes)	265	88.0
Regular use of a hand cream (no)	199	66.1
**Knowledge groups**		
Poor (<60% correct responses)	124	41.2
Moderate (60–79% correct responses)	174	57.8
Good (80–100% correct responses)	3	1.0

**Table 3:** Perception of healthcare workers about hand hygiene in Qassim, Saudi Arabia.

Perception	Frequency	Percent
**The impact of a healthcare-associated infection on a patient’s clinical outcome**		
Very low	1	0.3
Low	4	1.3
Neither high nor low	6	2.0
High	99	32.9
Very high	191	63.5
**The effectiveness of hand hygiene in preventing healthcare-associated infection**		
Very low	5	1.7
Low	2	0.7
Neither high nor low	7	2.3
High	83	27.6
Very high	204	67.8
**Among all patient safety issues, how important should hand hygiene be within your management priorities at your institution?**		
Very low priority	5	1.7
Low priority	4	1.3
Moderate priority	14	4.7
High priority	81	26.9
Very high priority	197	65.4
**Effectiveness of the following actions to increase hand hygiene permanently in a healthcare facility**		
**Leaders and senior managers support and openly promote hand hygiene**		
0 (Not effective)	5	1.7
1	4	1.3
2	17	5.6
3	107	35.5
4 (Very effective)	168	55.8
**The healthcare facility makes alcohol-based hand-rub always available at each point of care**		
0 (Not effective)	5	1.7
1	6	2.0
2	12	4.0
3	123	40.9
4 (Very effective)	155	51.5
**Hand hygiene posters are displayed at the point of care as reminders**		
0 (Not effective)	2	0.7
1	8	2.7
2	16	5.3
3	122	40.5
4 (Very effective)	153	50.8
**Each healthcare worker receives education on hand hygiene**		
0 (Not effective)	3	1.0
1	6	2.0
2	12	4.0
3	100	33.2
4 (Very effective)	180	59.8
**Clear and simple instructions for hand hygiene are made visible for every healthcare worker**		
0 (Not effective)	3	1.0
1	8	2.7
2	12	4.0
3	110	36.5
4 (Very effective)	168	55.8
**Healthcare workers regularly receive feedback on their hand hygiene performance**		
0 (Not effective)	4	1.3
1	14	4.7
2	48	15.9
3	110	36.5
4 (Very effective)	125	41.5
**Patients are invited to remind healthcare workers to perform hand hygiene**		
0 (Not effective)	12	4.0
1	72	23.9
2	69	22.9
3	62	20.6
4 (Very effective)	86	28.6
**Perception groups**		
Poor (<60% correct responses)	49	16.3
Moderate (60–79% correct responses)	221	73.4
Good (80–100% correct responses)	31	10.3

**Table 4:** Determinants of hand hygiene knowledge, perception, and practice among healthcare workers in Qassim, Saudi Arabia.

Factors	Moderate to Good Knowledge				Moderate to Good Perception				Routinely Use Alcohol-Based Hand-Rub			
	*p*	OR	95% CI for OR		*p*	OR	95% CI for OR		*p*	OR	95% CI for OR	
			Lower	Upper			Lower	Upper			Lower	Upper
**Gender**												
Female		1.00				1.00				1.00		
Male	0.044	0.52 *	0.28	0.98	0.461	0.71	0.28	1.78	0.065	0.28	0.07	1.08
**Age**												
20–34 years		1.00				1.00				1.00		
>34 years	0.062	0.55	0.30	1.03	0.716	1.18	0.49	2.86	0.321	1.97	0.52	7.49
**Profession**	0.190				0.760				0.266			
Technician		1.00				1.00				1.00		
Nurse	0.394	0.65	0.24	1.75	0.919	0.93	0.24	3.68	0.091	3.87	0.80	18.62
Dentist	0.409	1.83	0.44	7.68	0.656	0.65	0.10	4.28	0.998	-	-	-
Physician (MD)	0.872	1.09	0.40	2.93	0.933	1.06	0.26	4.25	0.042	4.90 *	1.06	22.75
Pharmacist	0.278	2.09	0.55	7.92	0.314	2.77	0.38	20.20	0.088	6.13	0.76	49.22
**Education**												
Graduate		1.00				1.00				1.00		
Postgraduate	0.162	0.53	0.22	1.29	0.018 *	0.27	0.09	0.80	0.000	0.09 *	0.03	0.31
**Nationality**												
Saudi		1.00				1.00				1.00		
Non-Saudi	0.072	2.03	0.94	4.38	0.341	1.69	0.58	4.93	0.552	0.64	0.15	2.76
**Types of healthcare facilities**												
Government		1.00				1.00				1.00		
Private	0.677	1.13	0.63	2.06	0.006 *	0.33	0.15	0.73	0.061	0.34	0.11	1.05
**Hand hygiene training**												
Did not receive		1.00				1.00				1.00		
Received	0.599	1.23	0.56	2.71	0.016 *	3.19	1.24	8.21	0.023	3.75 *	1.20	11.67

* statistically significant at *p* < 0.05 or *p* < 0.01.

## Data Availability

Data used in this study are available from the corresponding author on reasonable request.
